# The antibacterial activities of aditoprim and its efficacy in the treatment of swine streptococcosis

**DOI:** 10.1038/srep41370

**Published:** 2017-02-01

**Authors:** Guyue Cheng, Yamei Xu, Xudong Zhu, Shuyu Xie, Liye Wang, Lingli Huang, Haihong Hao, Zhenli Liu, Yuanhu Pan, Dongmei Chen, Yulian Wang, Zonghui Yuan

**Affiliations:** 1 National Reference Laboratory of Veterinary Drug Residues (HZAU) and MOA Key Laboratory for Detection of Veterinary Drug Residues, Huazhong Agricultural University, Wuhan, Hubei, 430070, China; 2 MOA Laboratory for Risk Assessment of Quality and Safety of Livestock and Poultry Products, Huazhong Agricultural University, Wuhan, Hubei, 430070, China

## Abstract

Aditoprim (ADP) has potential use as an antimicrobial agent in animals. However, its pharmacodynamic properties have not been systematically studied yet. In this study, the *in vitro* antibacterial activities of ADP and its main metabolites were assayed, and the *in vivo* antibacterial efficacy of ADP for the treatment of swine streptococcosis was evaluated. It was shown that *Salmonella* and *Streptococcus* from swine, *Escherichia coli* and *Salmonella* from chickens, *E. coli, Streptococcus, Mannheimia, Pasteurella* from calves, *Streptococcus* and *Mannheimia* from sheep, and *E. coli, Flavobacterium columnare, Acinetobacter baumannii* and *Yersinia ruckeri* from fishes were highly susceptible to ADP. *Haemophilus parasuis* from swine, *Staphylococcus aureus, Aeromonas punctate, Mycobacterium tuberculosis, Streptococcus agalactiae* from fishes, and *Klebsiella* from calves and sheep showed moderate susceptibility to ADP, whereas *E. coli, Actinobacillus pleuropneumonia, Pasteurella, S. aureus, Clostridium perfringens* from swine, *S. aureus, C. perfringens* from chickens, and *S. aureus* from calves were resistant to ADP. The main metabolites of ADP showed equal activity to that of their parent compound, and the prevention and therapeutic dosages of ADP recommended for swine streptococcosis were 10 and 20~40 mg/kg b.w., respectively. This study firstly showed that ADP had strong antibacterial activity and had potential to be used as a single drug in the treatment of bacterial infectious diseases.

Aditoprim (ADP, 2,4-diamino-5-(4-[dimethylamino]-3,5-dimethoxybenzyl)pyrimidine), is a dihydrofolate reductase inhibitor which inhibits the transformation of dihydrofolic acid to tetrahydrofolic acid^1^. The chemical structure of ADP is similar to that of another 2,4-diaminopyrimidine, trimethoprim (TMP), which demonstrates inhibitory activity for most gram-positive aerobic cocci and Gram-negative aerobic bacilli, especially *Staphylococcus aureus, Staphylococcus epidermidis*, Group A beta-hemolytic *streptococci, Streptococcus agalactiae, Streptococcus viridans, Streptococcus pneumoniae, Streptococcus faecalis, Escherichia coli, Klebsiella pneumoniae, Enterobacter* sp., *Salmonella* sp., *Shigella* sp., and *Hemophilus infIuenzae*^2^,^3^,^4^.

However, there are less studies on the pharmacodynamics of ADP at present. Early studies have demonstrated that ADP is a broad-spectrum drug, exhibiting antibacterial activity against many pathogens from food-producing animals, such as *Haemophilus, Enterobacteriaceae, Aeromonas, Vibrio, Staphylococci, Streptococci, Acinetobacter* and *Alcaligenes* (MICs ≤ 8 μg/mL, determined by using Diagnostic Sensitivity Test Agar)^1^. It is more active than sulfamethazine, sulfamethazine-TMP combination and tetracycline^1^. Another study using tube dilution method showed that the MICs of ADP were ranged from 5 to 20 μg/mL and the MBCs were ranged from 320 to 1280 μg/mL to *E. coli, Salmonella cholerasuis, Steptococcus, S. aureus*, and *Pasteurella multocida* (Li Shengbin, unpublished data). Since the methods used for MIC determination were not unified from different literatures, it is difficult to compare the results horizontally. Moreover, the kinds and numbers of bacteria were limited. Therefore, systematic research on the pharmacodynamics of ADP is urgently needed.

ADP has excellent pharmacokinetic characteristics. Previous studies have shown that ADP has longer elimination half lives (3.3~14.8 h) and higher distribution volumes (4.6~10.4 L/kg) than those of TMP in pig, calf, monkey, sheep and some other animal species^5^,^6^,^7^,^8^,^9^,^10^,^11^,^12^,^13^,^14^,^15^,^16^,^17^, and a similar pattern is observed for ADP where the distribution volume is about four times higher than that of TMP. For the poor pharmacokinetics^18^ and the development of antibacterial resistance^19^,^20^,^21^, TMP is often used in combination with sulfonamides as antibacterial synergist for the treatment and prevention of bacterial infections in veterinary and human medicine^22^. In addition, the general toxicity, the mutagenicity, the reproductive toxicity and the teratogenicity studies of ADP have demonstrated that it shows low toxicities^23^,^24^. These advantages make ADP a promising antibacterial agent in veterinary medicine.

ADP can be metabolized into 11, 9, 2 and 2 metabolites in swine, broilers, carp and rats, respectively, and *N*-monodesmethyl-ADP (A_1_) and *N*-didesmethyl-ADP (A_2_) are the main metabolites in their edible tissues^25^. In sheep, A_1_ and A_2_ also appear to be equally important metabolites^26^. Whether A_1_ or A_2_ has antibacterial activities, which is important for formulating the clinical dosage regimen, has not been studied.

Based on the related guidelines and standards of Clinical and Laboratory Standards Institute (CLSI), we determined the *in vitro* antibacterial activities of ADP, A_1_ and A_2_, and established the antimicrobial spectrum of ADP comprehensively and systematically in pathogenic bacteria from swine, chickens, fishes, calves and sheep in the present study. Phase II clinical trial was also carried out to evaluate the efficacy of ADP in the treatment of swine streptococcicosis. The deep knowledge about the pharmacodynamics of ADP will lay a solid foundation for the application of ADP as a new veterinary drug.

## Results

### The *in vitro* antibacterial activities of ADP and its metabolites

As shown in Table 1, 2 and 3, ADP has the same antibacterial spectrum as TMP. *Salmonella* and *Streptococcus* from swine, *E. coli* and *Salmonella* from chickens, *E. coli, Streptococcus, Mannheimia*, and *Pasteurella* from calves, *Streptococcus* and *Mannheimia* from sheep, and *E. coli, Flavobacterium columnare, A. baumannii* and *Y. ruckeri* from fishes were highly susceptible to ADP (MIC or MIC_50_ ≤ 4 μg/mL). *H. parasuis* from swine, *S. aureus, Aeromonas punctate, Mycobacterium tuberculosis*, and *S. agalactiae* from fishes, and *Klebsiella* from calves and sheep showed moderate susceptibility (MIC or MIC_50_ = 8~16 μg/mL), whereas *E. coli, A. pleuropneumonia, Pasteurella, S. aureus*, and *C. perfringens* from swine, *S. aureus* and *C. perfringens* from chickens, and *S. aureus* from calves were resistant to ADP (MIC or MIC_50_ ≥ 32 μg/mL). The values of MBC/MIC were from 1 to 4 for ADP against sensitive bacteria, which indicated that ADP had a good bactericidal activity.

ADP and TMP had equal *in vitro* activity against *E. coli, Salmonella* and *Streptococcus* from swine, and *F. columnare* from fishes. *Salmonella* from chicken, *E. coli, Streptococcus, Mannheimia*, and *Pasteurella* from calves, *Streptococcus* and *Mannheimia* from sheep, and *E. coli, A. punctate, A. baumannii, M. tuberculosis, S. agalactiae, S. aureus* and *Y. ruckeri* from fishes were more susceptible to ADP than to TMP, whereas the MICs and MBCs of ADP against *E. coli* from calves and chicken were slightly higher than those of TMP.

For those facultative anaerobes, the MIC and MBC of ADP for a single strain or the MIC_50_, MBC_50_, MIC_90_ and MBC_90_ for more strains were equal or differed by only one gradient (2-fold) in the anaerobic and aerobic conditions, indicating that ADP also showed strong antibacterial and bactericidal activity against facultative anaerobes in anaerobic conditions.

As shown in Table 4, the MIC_50_, MBC_50_, MIC_90_ and MBC_90_ of A_1_ and A_2_ against *Streptococcus* from swine and *Salmonella* from chickens were equal to those of the parent drug ADP, from which we can draw a conclusion that the *in vitro* antibacterial activities of ADP and its main metabolites, A_1_ and A_2_, are equally potent.

### Efficacy of ADP in the treatment of swine streptococcicosis

**Figure 1:**
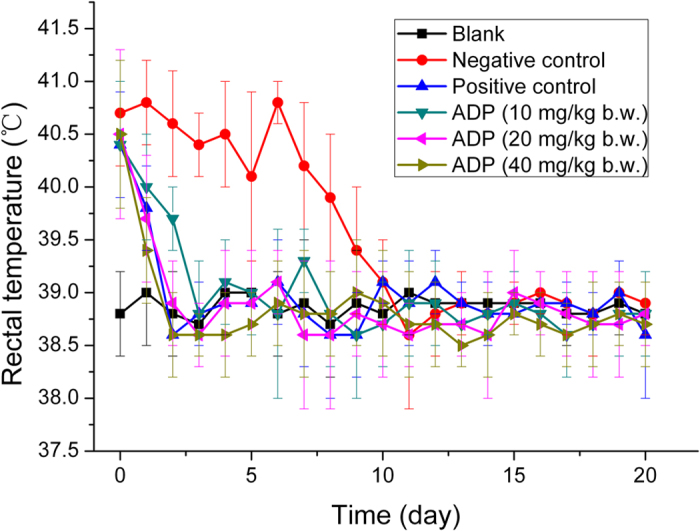
Changes in the mean rectal temperatures in each of the six groups of swine. The infected swine were treated once daily with indicated doses of ADP or Compound Sulfadiazine Sodium (Positive control) from Day 0 to Day 5. The average rectal temperatures of the healthy (Blank) and treated or untreated (Negative control) infected swine were observed and recorded from Day 0 to Day 20.

No clinical signs of streptococcicosis were seen in any of the swine during acclimation period prior to the infection challenge. During the treatment period, the mean rectal temperatures of pigs in the high-dose treatment group, the middle-dose treatment group and the positive group returned to normal level on the second day, and the mean temperature in the low-dose treatment group was back to normal level on the third day, whereas the temperature in the negative control group returned to normal on the ninth day (Fig. 1).

All swine in the infected groups showed lethargy and loss of appetite before their first dose, whereas about 30% and 60% of the swine developed limping and respiratory symptoms, respectively. Three swine in the negative control group developed symptoms of sliding on Day 2 and Day 3 and then died on the next day. Autopsy results showed a large amount of severe fibrino exudation on the serosa of lung and kidney, bleeding point on the liver and spleen and enlargement of mesenteric lymph nodes (Supplementary Fig. S1). The histopathological study revealed a brain neuron atrophy, splenic nodule reduction, polycythemia, and existence of inflammatory cells and a small number of erythropoiesis in lung. Modified particles of hepatocyte and glomeruli swollen were also found as shown in Supplementary Fig. S2.

Swabs from the center of the lung lesions from the dead swine appeared alpha-hemolysis on 5% sheep blood agar plates (Supplementary Fig. S3A). Selected individual bacterial colony was confirmed by PCR, which showed 498 bp and 566 bp DNA bands corresponding to the sizes of CPS-2J and GDH genes which are specific for *S. suis* serotype 2 and *Streptococcus*, respectively (Supplementary Fig. S3B). The above results indicated that the deaths of swine were caused by *S. suis* infection.

**Figure 2:**
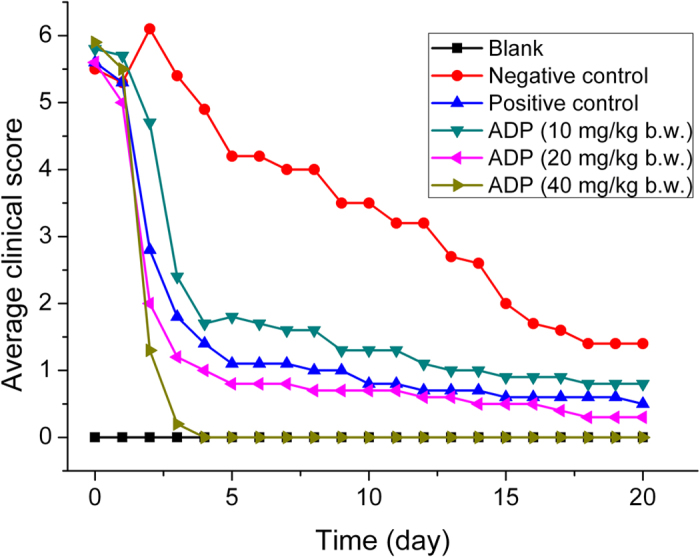
Changes in the mean clinical scores in each of the six groups of swine. The infected swine were treated once daily with indicated doses of ADP or Compound Sulfadiazine Sodium (Positive control) from Day 0 to Day 5. The average clinical scores of the healthy (Blank) and treated- or untreated- (Negative control) infected swine were observed and recorded from Day 0 to Day 20.

The symptoms of most of the swine in the treatment groups were back to normal after dosing of ADP on Day 2 of the high-dose treatment group and the middle-dose treatment group and on Day 3 of the Compound Sulfadiazine Sodium positive-control group and the low-dose treatment group. Three swine in the low-dose treatment group and one in the middle-dose treatment group usually showed moderate or mild cough and somnolence. Coughing was noted in two swine in the low-dose treatment and one in the middle-dose treatment group. The mean “clinical score” of per swine were showed in Fig. 2.

In order to evaluate the therapeutic effect of ADP on swine streptococcosis, a comprehensive analysis was done about coughing, mental state, body temperature and other symptoms of each group. As shown in Table 5, there were no deaths in the three ADP-treated groups, whereas the survival rate of the negative control group was 70%. The high-dose group of ADP showed 100% efficiency rate and cure rate, while both of the effective rate and the cure rate of the middle-dose group and the positive drug control group were 90%. The cure rate and the effective rate of the low-dose group were 80% and 70%, respectively (Table 5).

The body weights of the swine in each group were tested and compared before and after the experiment (Table 6). The relative weight gain rate (RAW) of Compound Sulfadiazine Sodium control group (93.0%) was between those of the high-dose treatment group (96.5%) and the middle-dose treatment group (91%), without significant difference between each other (P > 0.05). In the low-dose treatment group, the RAW was 74.7%, which is significantly different when compared with those of the high-dose group and the middle-dose group (P < 0.05). Swine in the negative control group gained an average weight of only 1.77 kg with RAW of 34.5%, showing a significant difference with those of the high-dose, the middle-dose and the low-dose groups (P < 0.05).

## Discussion

As a newly developing veterinary drug, ADP has turned out to be low toxic and not mutagenic^23^,^24^. It exhibits longer half-life in animals than the representative drug of 2, 4-diaminopyrimidines, TMP, probably due to the dimethylamino group, which is vital for lengthened elimination rate in this compound^27^. In this study, the antimicrobial activities of ADP against zoonotic pathogens were investigated systematically and were compared with TMP. Results showed that ADP had a similar degree of *in vitro* antibacterial activities as those of TMP. Many gram-positive and gram-negative bacteria are highly susceptible or susceptible to ADP, especially *Salmonella* and *Streptococcu*s, while *H. parasuis* and *Klebsiella* were moderately susceptible to ADP. Previous study showed that *Pseudomonas aeruginosa, Campylobacter spp, Actinomyces* and *Clostridium spp.* presented natural resistance to TMP and ADP^1^, which was validated in this study. In contrast, *S. aureus* are resistant to ADP and TMP in our study, which may be due to the single mutation (Phe98 to Tyr98) in the dihydrofolate reductase of *S. aureus*^28^,^29^,^30^. Interestingly, we found that ADP presents high *in vitro* antibacterial activities against pathogenic bacteria from calves and sheep like baquiloprim^31^.

Antibacterial resistance is a serious problem which threatens human health in recent times, so it is very important for us to improve the rational use of drugs in order to prevent the development of resistance. However, we often overlook the antibacterial activity of the drug metabolites, which is a critical factor in formulating dosage regimen. Twelve metabolites of ADP can be detected in swine, broilers, carp and rats, and A_1_ and A_2_, which occupied 11.4% and 11.8% in swine, 12.8% and 14.0% in broilers, respectively, were the two main metabolites in these edible tissues^25^. In this study, the *in vitro* activities of A_1_ and A_2_ on *Streptococcus* from swine and *Salmonella* from chickens were equal to those of ADP. Therefore, we can have an inference that ADP would exhibit strong and long-lasting *in vivo* antibacterial activity when A_1_ and A_2_ are considered.

TMP, with a short half-life in domestic animals, presents shortcomings in maintaining sufficient plasma and tissue concentrations, so it is often used in combination with sulfonamides^32^,^33^,^34^,^35^,^36^,^37^. In contrast to TMP, the superior pharmacokinetic properties of ADP allowed it as a single agent for therapeutic application. In this study, the *in vivo* antibacterial activity of ADP was determined by evaluating its efficacy in swine infected by *S. suis* type 2 CVCC607, which is a standard isolates and whose MIC is closed to the MIC_50_ of ADP to *S. suis*. As there were no recommended dose of ADP in veterinary clinic, administration dosage was set according to the pharmacodynamic and pharmacokinetic properties of ADP. It is generally accepted that in order to achieve a therapeutic effect, the plasma concentration of an antimicrobial should be higher or equal to the MIC for approximately half of the dosing time-interval of the drug^38^. Consequently, based on the MIC of ADP against *S. suis* CVCC607 and the pharmacokinetic values calculated after injection of ADP intramuscularly (Du Xi, unpublished data), 10 mg/kg b.w., 20 mg/kg b.w. and 40 mg/kg b.w. were set as the high, the medium and the low dosage groups, respectively, with once-daily administration.

Results of changes in rectal temperature, mental state, claudication and respiratory symptom indicated that all dosage groups of ADP exhibited therapeutic action to a certain extent, when compared with negative group. The efficacies of the high-dose treatment group and the middle-dose treatment group were better than that of the low-dose treatment group, while the efficacy of the middle-dose treatment group were equal to that of the positive control group. Therefore, the therapeutic dose of ADP recommended for swine streptococcosis is 20–40 mg/kg b.w., while 10 mg/kg b.w. can be used for prevention or consolidating its curative effect. Since 2, 4-diaminopyrimidines are often used as antibacterial synerists, there was no recommended dose for monotherapy in clinic. The combined dose of 40 mg/kg b.w. baquiloprim and sulphadimidine was the minimum recommended dosage for use as bolus in the field^32^. The oral suspension of TMP plus sulfadiazine administered at 24 mg/kg b.w. twice daily effectively treated the clinical signs of *S. equi subsp zooepidemicus* lower respiratory infection in horses^33^. In this study, the clinical efficacy of the single preparation of ADP is equivalent or better than those of the other 2, 4-diaminopyrimidine compound preparations.

In conclusion, this study has determined MICs and MBCs of ADP against common pathogens from swine, chickens, fishes, calves and sheep and established the antibacterial spectrum of ADP. It is shown that ADP has a good antibacterial activity which is identical with TMP, so do its main metabolites, A_1_ and A_2_. Phase II clinical study of ADP on therapeutic effect on swine streptococcosis has demonstrated that ADP is as active as compound sulfadiazine, which provides reasonable theoretical foundation for the clinical application of ADP. The overall *in vitro* and *in vivo* results provide predictive evidence that ADP has high antibacterial activity that can be used alone even though we are hunting appropriate medications for drug combinations.

## Materials and Methods

### Bacteria

*E. coli* ATCC25922, *S. aureus* ATCC29213, *Enterococcus faecalis* ATCC29212, *Pseudomonas aeruginosa* ATCC27853 and *Streptococcus pneumonia* ATCC49619 were obtained from America Type Culture Collection and were used as quality control strains. Standard strains such as *E. coli* CVCC220, CVCC1496, CVCC1500, CVCC 1502, CVCC 1513, CVCC1519 and CVCC2083, *Salmonella typhimurium* CVCC542 and CVCC541, *Clostridium perfringens* CVCC1125, CVCC1160 and CVCC2030, and *Streptococcus suis* CVCC607 and CVCC609 were purchased from Chinese Veterinary Culture Collection (Beijing, China). Isolated strains from swine, chickens, fishes, calves and sheep in livestock farms were also used in this study.

### Animals and ethic statement

Sixty 3-week-old swine weighing 11 to 13 kg, which were completely healthy with no history of streptococcosis, were purchased from Jinlong Livestock and Poultry Co., Ltd (Wuhan, China). The animal houses were comfortable with standard temperature and humidity. They were free to access water and fed with a commercial diet free of antibiotics twice daily. All swine were allowed 7-day acclimation before the study. All the animal experiment procedures were performed in accordance with the guidelines and regulations of Animal Care Center, Hubei Science and Technology Agency in China (SYXK 2013-0044), and the experimental protocols were approved by the Ethics Committee of Huazhong Agricultural University, Wuhan, China.

### Chemicals

ADP and its metabolites, A_1_ and A_2_, with chemical purity >99% were synthesized at National Reference Laboratory of Veterinary Drug Residues, Huazhong Agricultural University (Wuhan, China). TMP was purchased from China Institute of Veterinary Drugs Control (Beijing, China). Sulfadiazine Sodium Injection was bought from Zhengzhou Bairui Animal Pharmaceutical Co., LTD (Zhengzhou, China).

### Antimicrobial susceptibility testing

Minimum inhibitory concentrations (MICs) of ADP and its main metabolites were determined by the broth microdilution technique according to the CLSI guidelines^39^,^40^,^41^. Minimum bactericidal concentrations (MBCs) was determined as follows. After MIC reading, 10 μL bacteria suspension from wells in which the drug concentrations were higher than MIC were plated on agar and incubated overnight. MBC was the lowest concentration which could achieve 99.9% killing^42^. Using SPSS 19.0, the 50% minimum inhibitory concentration (MIC_50_), 90% minimum inhibitory concentration (MIC_90_), 50% minimum bactericidal concentration (MBC_50_) and 90% minimum bactericidal concentration (MBC_90_) were calculated.

### The establishment of swine infectious model with *Streptococcus*

According to the results of *in vitro* antibacterial activity of ADP, *S. suis* CVCC607 was used for evaluating the therapeutic effect of ADP on swine streptococcicosis. The swine were randomly allocated into six groups (n = 10 for each group). Ten healthy swine were inoculated subcutaneously with 2 mL normal saline and were set as the blank group. Swine in the other five groups were inoculated subcutaneously with 2 mL inoculum containing 10^10^ CFU of *S. suis* CVCC607. The construction of model was successful when 70% of the swine gained the typical symptoms of streptococcicosis. To prevent cross-contamination, animals were housed in six different experimental rooms.

### Treatment protocol

Treatment was applied upon streptococcicosis infection swine model. Swine in the negative-control group were monitored without drug treatment and swine in the positive-control group were treated with Compound Sulfadiazine Sodium (30 mg/kg b.w.). Swine in the low-dose treatment group, the middle-dose treatment group and the high-dose treatment group were treated with ADP injection at doses of 10 mg/kg b.w., 20 mg/kg b.w., and 40 mg/kg b.w., respectively. All the treatment groups were administered once daily for 5 days and observed for 15 days after the first administration.

### Clinical observations

Health observations like mental states, appetite, rectal temperature and other clinical symptoms were recorded every day. The presences of lethargy, loss of appetite, claudication and respiratory abnormality (coughing or panting) were scored as follows in order to make a daily “clinical score”. Breath state: score 0, normal; score 1, increased breathing rate or occasional coughing; score 2, abdominal breathing or regular coughing; score 3, showing signs of severe dyspnea. Mental state: score 0, normal; score 1, mild depression; score 2, usually lying down or standing when gentle stimulated; score 3, holding up on forelegs in a sitting position or markedly depressed. Cure was defined when the rectal temperature was below 39.5 °C and both mental state score and breath state score were ≤1, while a medical improvement of the disease were considered effective^43^,^44^.

Autopsy was carried out on the dead animals. Tissues were fixed with 10% formalin, dehydrated, immersed in the wax, cut into slices, and stained with hematoxylin eosin in order to observe the pathological changes of tissues. Swabs from the center of lesions were streaked on 5% sheep blood agar plates to make the bacteriological examination. Selected individual bacterial colony were examined using primers designed to amplify specific genes (5′-TTTGTCGGGAGGGTTACTTG-3′ and 5′-TTTGGAAGCGATTCATCTCC-3′ for CPS-2J gene; 5′-AAGTTCCTCGGTTTTGAGCA-3′ and 5′-GCAGCGTATTCTGTCAAACG-3′ for GDH gene) of *S. suis* serotype 2 and *Streptococcus* to make sure that the deaths were caused by *S. suis* serotype 2 infections. Mortality was recorded during the whole experiment and relative weight gain rate (RAW) was calculated through individual bodyweights at the beginning and finish of the study.

### Statistical analysis

MIC_50_, MBC_50_, MIC_90_ and MBC_90_ were calculated by using SPSS software. Statistical analysis was performed with Student’s t-test and Bonferroni revision for comparing the clinical variables before and after treatment, and differences were considered to be statistically significant when the p-value was less than 0.05.

## Additional Information

**How to cite this article**: Cheng, G. *et al*. The antibacterial activities of aditoprim and its efficacy in the treatment of swine streptococcosis. *Sci. Rep.* **7**, 41370; doi: 10.1038/srep41370 (2017).

**Publisher's note:** Springer Nature remains neutral with regard to jurisdictional claims in published maps and institutional affiliations.

## Supplementary Material

Supplementary Figures

## Figures and Tables

**Table 1:** The MIC_50_, MBC_50_, MIC_90_ and MBC_90_ of ADP and TMP against common pathogenic bacteria isolated from swine, chickens and calves.

Origin	Strains (amount)	MIC_50_ (μg/mL)		MBC_50_ (μg/mL)		MBC_50_/MIC_50_		MIC_90_ (μg/mL)		MBC_90_ (μg/mL)		MBC_90_/MIC_90_	
		ADP	TMP	ADP	TMP	ADP	TMP	ADP	TMP	ADP	TMP	ADP	TMP
Swine	*E. coli* (53)	64	64	128	128	2	2	>128	>128	>128	>128	—	—
	*Salmonella* (43)	2	2	8	8	4	4	64	64	128	128	2	2
	*Streptococcus* (44)	2	2	4	2	2	1	32	32	64	128	2	4
	*A. pleuropneumonia* (12)	128	>128	>128	>128	—	—	128	>128	>128	>128	—	—
	*H. parasuis* (31)	16	8	32	8	2	1	32	64	64	128	2	2
	*Pasteurella* (9)	64	64	128	128	2	2	64	>128	128	>128	2	—
Chicken	*E. coli* (33)	1	0.5	4	2	4	4	>128	128	>128	>128	—	—
	*Salmonella* (43)	1	1	2	4	2	4	8	8	16	16	2	2
Calves	*E. coli* (20)	4	2	16	8	4	4	16	16	128	128	8	8
	*S. aureus* (16)	128	128	>128	>128	—	—	>128	>128	>128	>128	—	—

**Table 2:** The MICs and MBCs of ADP and TMP against common pathogenic bacteria isolated from swine, chickens, calves and sheep.

Origin	Strains	Number	ADP		TMP	
			MIC (μg/mL)	MBC (μg/mL)	MIC (μg/mL)	MBC (μg/mL)
Swine	*S. aureus*	207	32	128	16	32
	*S. aureus*	126b	32	128	16	64
	*S. aureus*	1256	64	128	128	>128
	*S. aureus*	586	64	>128	>128	>128
	*C. perfringens*	46P	>128	>128	64	>128
	*C. perfringens*	CVCC1160	>128	>128	>128	>128
	*C. perfringens*	45P	>128	>128	64	>128
	*C. perfringens*	55	>128	>128	>128	>128
	*C. perfringens*	F12P	>128	>128	64	>128
Chickens	*S. aureus*	107	64	>128	32	64
	*S. aureus*	12	>128	>128	64	>128
	*C. perfringens*	CVCC2030	>128	>128	>128	>128
	*C. perfringens*	12	>128	>128	>128	>128
Calves	*Streptococcus*	444	4	8	4	8
	*Streptococcus*	689	2	2	2	4
	*Pasteurella*	Hunag 4.26	2	2	2	4
	*Pasteurella*	Huang A	32	64	32	64
	*Pasteurella*	Stander B	32	128	32	128
	*Pasteurella*	16	4	8	4	8
	*Klebsiella*	145	8	32	8	32
	*Klebsiella*	350	4	8	4	16
	*Klebsiella*	340	16	64	16	64
Sheep	*Mannheimia*	MCMH	0.5	2	0.25	1
	*Mannheimia*	MH0529	0.5	2	0.25	1
	*Pasteurella*	1064	1	2	1	4
	*Pasteurella*	Hong 4.26	4	8	4	8
	*Klebsiella*	292	8	32	8	32
	*Klebsiella*	284	8	32	8	32
	*Klebsiella*	294	8	32	8	32

**Table 3:** The MICs and MBCs of ADP and TMP against common pathogenic bacteria isolated from fishes.

Strains	Number	ADP		TMP	
		MIC (μg/mL)	MBC (μg/mL)	MIC (μg/mL)	MBC (μg/mL)
*Y. ruckeri*	SC90-2-4	0.5	1	4	4
*E. coli*	Se-1	0.5	1	1	2
*Aeromonas punctata*	58-20-9	8	16	>128	>128
*Aeromonas hydrophils*	Ah563	32	128	128	>128
*Aeromonas hydrophils*	Ah561	128	128	>128	>128
*Aeromonas hydrophils*	Ah78	128	128	>128	>128
*Aeromonas hydrophils*	SX91-4-1	128	128	>128	>128
*Aeromonas caviae*	DMA1-A	128	128	>128	>128
*Aeromonas sobria*	CR79-1-1	128	128	>128	>128
*Aeromonas veronii*	ATCC9071	128	128	>128	>128
*Aeromonas jandaei*	F30-3	128	128	>128	>128
*Pseudomonas fluorescent*	W81-11	128	128	>128	>128
*Pseudomonas fluorescent*	56-12-10	128	128	>128	>128
*Flavobacterium columnare*	G4	2	4	2	4
*Edwardsiella ictaluri*	HSN-1	128	128	>128	>128
*Streptococcus agalactiae*	XQ-1	8	16	8	16
*S. aureus*	Fs1	32	64	32	64
*S. aureus*	Fs2	8	16	16	32
*M. tuberculosis*	Asc-1.2 II	4	8	8	8
*M. tuberculosis*	Asc-1.3 II	8	16	8	32
*M. tuberculosis*	Asc-1.3V	8	16	8	16
*M. tuberculosis*	Cst-t-10	16	64	32	64
*A. baumanii*	Ab1	128	128	>128	>128

**Table 4:** The MIC_50_, MBC_50_, MIC_90_ and MBC_90_ of ADP and its metabolites against *Streptococcus* from swine and *Salmonella* from chickens.

	**ADP**	**A1**	**A2**
Streptococcus from swine			
MIC_50_ (μg/mL)	2	2	2
MBC_50_ (μg/mL)	4	4	4
MBC_50_/MIC_50_	2	2	2
MIC_90_ (μg/mL)	32	32	32
MBC_90_ (μg/mL)	64	64	64
MBC_90_/MIC_90_	2	2	2
Salmonella from chickens			
MIC_50_ (μg/mL)	1	1	1
MBC_50_ (μg/mL)	2	2	2
MBC_50_/MIC_50_	2	2	2
MIC_90_ (μg/mL)	8	8	8
MBC_90_ (μg/mL)	16	16	16
MBC_90_/MIC_90_	2	2	2

**Table 5:** The efficacy of ADP in the treatment of swine streptococcicosis.

Group (n = 10)	Survival rate (%)	Mortality rate (%)	Effective rate (%)	Cure rate (%)
Blank	100	0	—	—
Negative control	70	30	—	—
Positive control	100	0	90	90
Low-dose treatment group (10 mg/kg b.w.)	100	0	80	70
Middle-dose treatment group(20 mg/kg b.w.)	100	0	90	90
High-dose treatment group (40 mg/kg b.w.)	100	0	100	100

**Table 6:** Changes of the mean group weight.

Group (n = 10)	Initial weight (kg)	Final weight (kg)	Net gain (kg)	RWG (%)
Blank	12.29 ± 0.62	17.42 ± 1.11	5.13	100.0^a^
Negative control	12.59 ± 0.64	14.36 ± 0.69	1.77	34.5^c^
Positive control	12.40 ± 0.60	17.17 ± 0.84	4.77	93.0^a^
Low-dose treatment group (10 mg/kg b.w.)	12.51 ± 0.69	16.34 ± 0.93	3.83	74.7^b^
Middle-dose treatment group (20 mg/kg b.w.)	12.47 ± 0.51	17.14 ± 0.70	4.67	91.0^a^
High-dose treatment group (40 mg/kg b.w.)	12.56 ± 0.76	17.51 ± 0.54	4.95	96.5^a^

RWG: relative weight gain rate. ^a,b,c^Statistically significant differences between groups indicated by different letters (P < 0.05).
