# Supplementary material for: The antibacterial activities of aditoprim and its efficacy in the treatment of swine streptococcosis
Source: Sci Rep. 2017 Feb 1;7:41370. doi: 10.1038/srep41370 (PMC5286432; doi:10.1038/srep41370)
Supplement: Supplementary Figures [file srep41370-s1.pdf]

Supplementary data

The antibacterial activities of aditoprim and its efficacy in the treatment of swine streptococcosis

Guyue Cheng [a, #], Yamei Xu [b, #], Xudong Zhu[b], Shuyu Xie[a], Liye Wang[b], Lingli Huang[a], Haihong Hao[a], Zhenli Liu[a], Yuanhu Pan[a], Dongmei Chen[a], Yulian Wang[a,]*, Zonghui Yuan[a,b,]*

Affiliations:

[a]National Reference Laboratory of Veterinary Drug Residues (HZAU) and MOA Key Laboratory for Detection of Veterinary Drug Residues, Huazhong Agricultural University, Wuhan, Hubei, 430070, China

[b]MOA Laboratory for Risk Assessment of Quality and Safety of Livestock and Poultry Products, Huazhong Agricultural University, Wuhan, Hubei, 430070, China

[#] Guyue Cheng and Yamei Xu contributed equally to this work.

***Corresponding author:**

Associated Prof. Dr. Yulian Wang, E-mail: wangyulian@mail.hzau.edu.cn

Prof. Dr. Zonghui Yuan, E-mail: yuan5802@mail.hzau.edu.cn

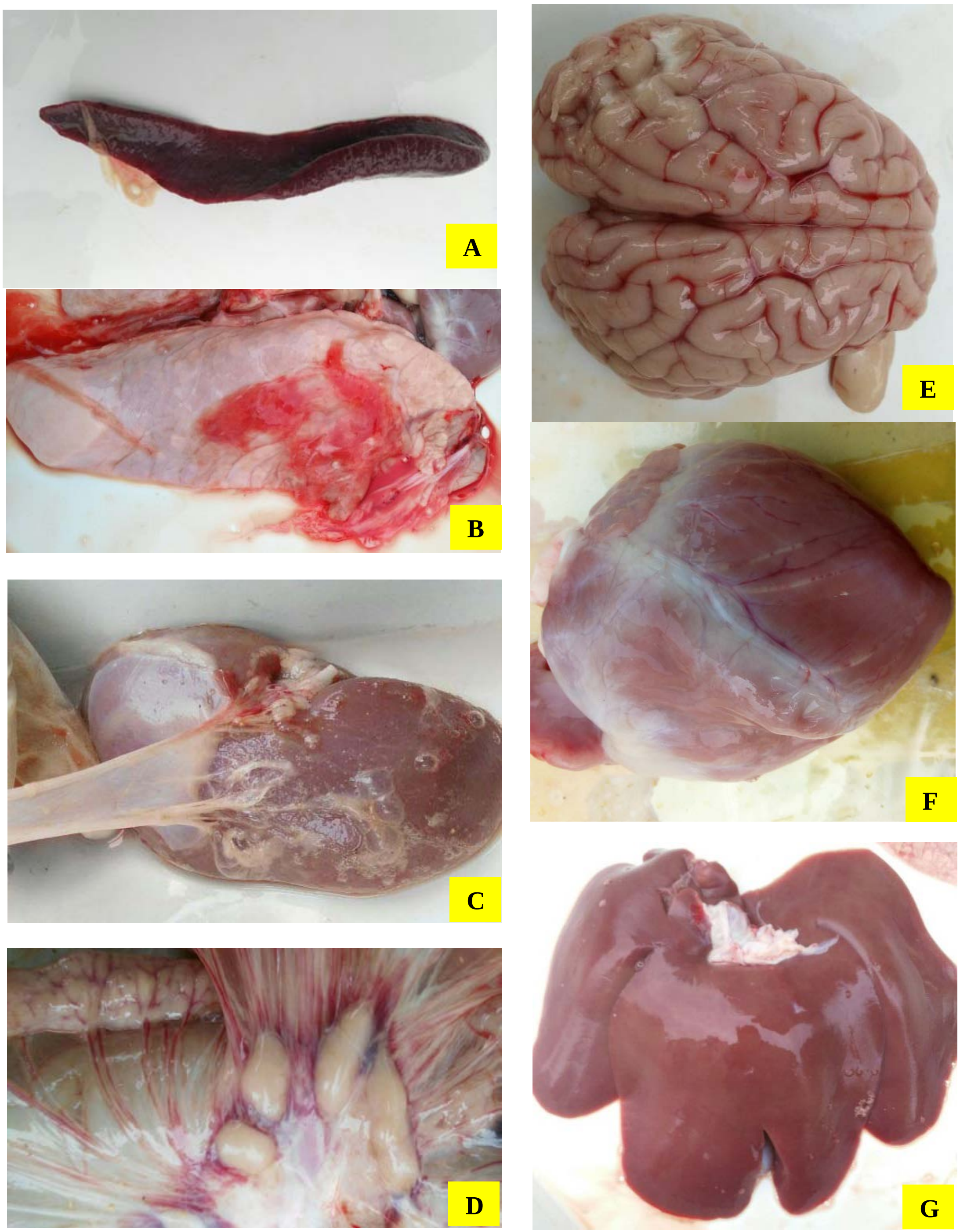

Supplementary Fig. S1. Pathological anatomy examination of dead swine infected with *S. suis* CVCC607. (A) Hemorrhage and necrosis of spleen; (B) Cellulose adhesion of lung; (C) Cellulose adhesion of kidney; (D) Lymph node enlargement; (E) Meninges bleeding; (F) Normal heart; (G) Hepatomegaly.

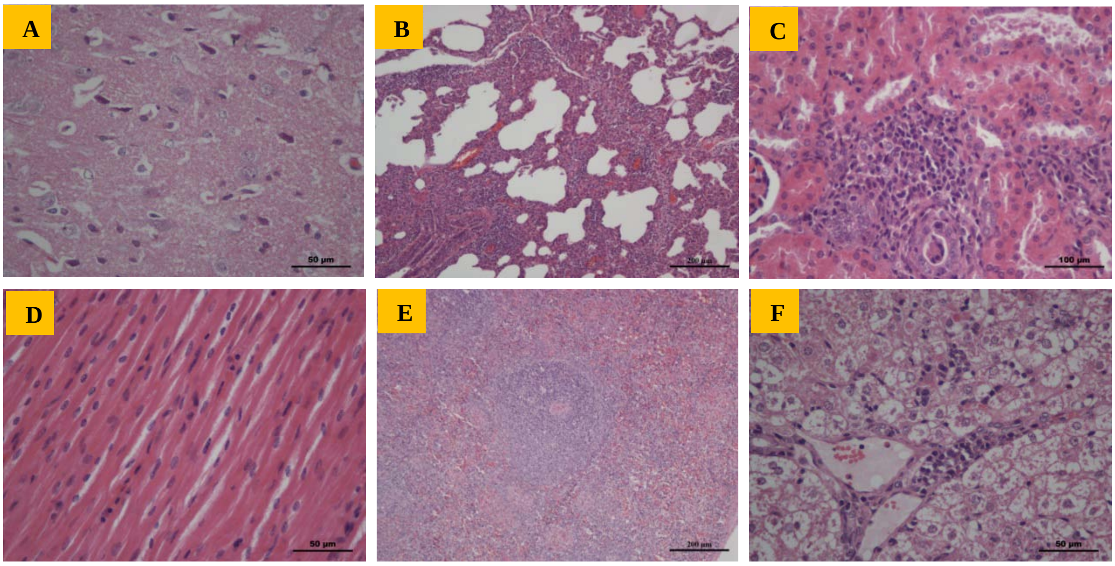

Supplementary Fig. S2. Pathological changes of dead swine infected with *S. suis* CVCC607. (A) Brain neuron atrophy; (B) Inflammatory cells, red blood cell and interstitial pneumonia; (C) Glomerular volume became bigger with epithelial degeneration; (D) Normal heart; (E) Splenic nodule reduction and polycythemia; (F) Flocular degeneration and vacuolar degeneration of liver cells.

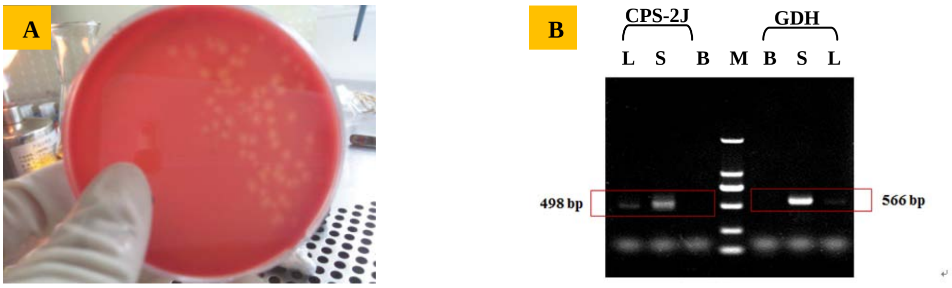

Supplementary Fig. S3. *S. suis* identification in the swabs from the center of lung lesions of swine infected with *S. suis* CVCC607. (A) Swabs from the center of lung lesions appeared the character of alpha-hemolysis on the 5% sheep blood agar plates. (B) Selected individual bacterial colony was confirmed by PCR, which showed 498bp and 566bp DNA bands corresponding to the sizes of the genes of CPS-2J and GDH specific for *S. suis* serotype 2 and *Streptococcus* respectively (“L” represents “Lung”, “S” represents “***S. suis*** CVCC607”, “B” represents “Blank”, and “M” represents “DNA marker”).
